# Supplementary material for: Absolute Quantitation of Serum Antibody Reactivity Using the Richards Growth Model for Antigen Microspot Titration
Source: Sensors (Basel). 2022 May 23;22(10):3962. doi: 10.3390/s22103962 (PMC9147899; doi:10.3390/s22103962)
Supplement: Supplementary file 1 [file sensors-22-03962-s001.zip › supplementary_textS2.pdf]

Supplementary text 2

Interpretation of the logarithmic form of the Richards curve as titration of chemical potential

Chemical potential μ by definition is

$$\mu=\mu°+\ln(a) \qquad \text{(S1)}$$

where μ° is standard chemical potential, *a* is relative thermodynamic activity:

$$a=c\gamma \qquad \text{(S2)}$$

where *c* is molar concentration and γ is activity coefficient.

The chemical potential of a given antibody species is then described, after replacing *a* in (S1) with γ*c* from (S2)

$$\mu_{Ab} = \mu°_{Ab} + \ln(c_{Ab}\gamma_{Ab}) \qquad \text{(S3)}$$

$$\mu_{Ab} = \mu°_{Ab} + ln(c_{Ab}) + \ln(\gamma_{Ab}) \qquad \text{(S4)}$$

Omitting the standard term and raising both sides to the exponent, we obtain the proportionality

$$e^{\mu_{Ab}} \sim e^{\ln(c_{Ab})+\ln(\gamma_{Ab})} \qquad \text{(S5)}$$

We assume that the fluorescence intensity signals are a measure of the absolute thermodynamic activity of antibodies, λ_{Ab}

$$FI_{Ab} \sim \lambda_{Ab} = e^{\mu_{Ab}} \qquad \text{(S6)}$$

Therefore, we suggest that in the logarithmic form of Richards function with k=1

$$\ln(FI_{Ab)} = \ln(A) + \ln((1 + (d-1)e^{-(x-x_i)})^{\frac{1}{1-d}}) \qquad \text{(S7)}$$

ln(A) is the logarithm of fluorescent intensity corresponding to the detection antibody with molar concentration $ln(c_{Ab})$ in S5, and the rest of the equation corresponds to logarithm of activity coefficient of antibody $\ln(\gamma_{Ab})$. However, we also propose that this second part of the equation also comprises activity coefficients and molar concentrations.

Distributing the negative sign in the exponent in S7, we get

$$\ln(FI_{Ab}) = \ln(A) + \ln((1 + (d-1)e^{x_i-x})^{\frac{1}{1-d}}) \qquad \text{(S8)}$$

then assuming 1/(d-1)=γ_{Ag}^{∞} (S9)

where γ_{Ag}^{∞} is constant for a given interaction at infinite Ab dilution, we obtain

$$\ln(FI_{Ab)} = \ln(A) + \ln((1 + \frac{1}{\gamma_{Ag}^{\infty}} e^{x_i-x})^{-\gamma_{Ag}^{\infty}}) \qquad \text{(S10)}$$

By substituting e^x=[Ag] and e^{xi}=$[Ag]_i$

$$\ln(FI_{Ab)} = \ln(A) + \ln((1 + \frac{1}{\gamma_{Ag}^{\infty}} \frac{[Ag]_i}{[Ag]})^{-\gamma_{Ag}^{\infty}}) \qquad \text{(S11)}$$

Comparing S4 and S11, the proposed activity coefficient of the measured antibody (highlighted in yellow) changes as a function of Ag concentration at inflection point $[Ag]_i$, concentration of antigen [Ag], and antigen activity coefficient γ_{Ag}^{∞} at infinite antibody dilution.

Overall, chemical potentials of the interaction partners are interdependent and are determined by the affinity distribution of antibodies in our measurement system.
